# Bacterial Volatile Isovaleric Acid Triggers Growth Alteration of *Arabidopsis* Seedlings

**DOI:** 10.3390/metabo12111043

**Published:** 2022-10-30

**Authors:** Jun Murata, Takehiro Watanabe, Hajime Komura

**Affiliations:** 1 Division of Integrative Biomolecular Function, Institute for Bioorganic Research, Suntory Foundation for Life Sciences, 8-1-1 Seikadai, Seika, Soraku, Kyoto 619-0284, Japan; 2 Research Planning Division, Institute for Bioorganic Research, Suntory Foundation for Life Sciences, 8-1-1 Seikadai, Seika, Soraku, Kyoto 619-0284, Japan; 3 Division of Structural Biomolecular Science, Institute for Bioorganic Research, Suntory Foundation for Life Sciences, 8-1-1 Seikadai, Seika, Soraku, Kyoto 619-0284, Japan

**Keywords:** bacterial volatile organic compounds, plant–microbe interaction, growth regulation, *Arabidopsis thaliana*, *Bacillus* spp.

## Abstract

Bacterial volatile organic compounds (BVOCs) released from selected soil microbes have been shown to trigger the alteration of plant growth. However, the substances responsible for such bioactivity and the mechanism of how plants interpret and respond to BVOCs remain largely elusive. Here, we established a model bioassay system using *Arabidopsis* and *Bacillus* spp. and found that *Bacillus* BVOCs interfere with the normal growth of *Arabidopsis* seedlings. Moreover, through a bioassay-guided purification, we identified isovaleric acid (IVA) as a volatile compound that exhibits inhibitory growth activity towards *Arabidopsis* seedlings. Our data provide novel molecular insights into how short-chain fatty acids released from soil microbes can affect plant growth through interkingdom signals.

## 1. Introduction

Plants and soil microbes intimately associate and mutually affect their respective fitness for survival in the rhizosphere [1]. Among the various modes of plant–microbe communication, one of the best studied examples is the symbiosis between legumes and diazotrophic rhizobia; carbon, which is fixed through photosynthesis by the legumes, is supplied to the rhizobia; in turn, nitrogen, which is fixed from the atmosphere by the rhizobia, is transported to the legumes [2]. The key signaling molecules in this association are the rhizobial lipochitooligosaccharides known as Nod factors, which are recognized by legume LysM receptor-like kinases and induce downstream events, including the development of infection threads. On the other hand, selected soil microbes are known to produce phytohormones (i.e., auxins) and are thus able to modulate plant growth [3,4]. However, the molecular basis for establishing the communication between plants and the vast majority of soil microbes is still largely unclear. This is especially the case with plant–microbe communication via so-called bacterial volatile organic compounds (BVOCs) from soil bacteria [5,6]. BVOCs that are produced by soil bacteria are released into the rhizosphere as well as the atmosphere at substantial levels and have a potential impact on global climate change [7]. BVOCs have also been studied in relation to their ability to alter the behavior and growing fitness of various organisms, including plants [8,9]. Recent studies demonstrate that specialized soil bacteria called plant growth-promoting rhizobacteria (PGPR) emit BVOCs that trigger plant growth promotion. However, only very few reports have identified BVOCs that are responsible for elaborating the bioactivity of a bacterial species through activity-guided purification. A pioneering work by Ryu et al. showed that BVOCs released from *Bacillus subtilis* GB03 and *B. amyloliquefaciens* IN937a were able to promote the growth of *Arabidopsis thaliana*, and their plant growth promoting activity was correlated well with the release of 2,3-butanediol, which was not detected from bacterial strains without such plant growth-promoting activity [10]. More recently, dimethyl disulfide from *Bacillus* sp. B55 was shown to promote growth in *Nicotiana* plants [11]. In both cases, however, the soil bacteria used in the studies were specialized strains with unique features as PGPRs. Therefore, whether there are BVOCs that function as plant growth regulators in other combinations of plant–soil bacteria interactions still must be investigated further. Moreover, because the soil bacteria that have been tested as emitters of BVOCs that affect plant growth have been limited to PGPRs [12,13,14], not much has been elucidated as to whether there are BVOCs that exhibit negative, rather than positive, impact upon plant growth.

In this study, we identify a novel mode of plant–microbe interaction between *A. thaliana* (Col-0) and *B. atrophaeus* Nakamura ATCC9372 (renamed from *B. subtilis*, ATCC9372) through BVOCs that trigger growth inhibition. We conducted activity-guided purification of the volatile compounds from the *B. atrophaeus* cells and identified that isovaleric acid is a key molecule that is able to induce plant growth alterations among a blend of BVOCs released from *B. atrophaeus*. Our results highlight the biological importance of isovaleric acid as a novel plant growth regulator and give insight into how plants and soil bacteria achieve interkingdom signaling through BVOCs that are ubiquitously expressed throughout bacterial species.

## 2. Materials and Methods

### 2.1. Culture of Plant Seedlings and Bacillus spp.

Plant seedlings were aseptically pre-cultivated prior to all bioassays. *A. thaliana* (Col-0) seeds were surface-sterilized, sowed on half-strength Murashige–Skoog medium [15] with 0.8% Phytoblend (Caisson laboratories, Smithfield, UT, USA), and cultivated under a long-day photoperiod (16-h-light/8-h-dark cycle) at 22 °C for 5 days. The agar plates (rectangular, 9 × 15 cm) were placed vertically in the growth chamber for easier visual inspections and measurements of the length of seminal roots. *B. atrophaeus* ATCC9372 was pre-cultured in 3 mL of tryptic soy (TS) liquid medium at 30 °C overnight and diluted 4-fold with fresh TS liquid medium before the designated volume of the culture was subjected to bioassays.

### 2.2. Chemicals

All the chemicals were purchased from Nacalai Tesque (Kyoto, Japan), Tokyo Chemical Industry (Tokyo, Japan), and Sigma Aldrich (St. Louis, MO, USA) unless otherwise stated.

### 2.3. Bioassays

The bioassays for detecting plant growth-regulating activity were performed in a rectangular plate that contained a round 35 mm petri dish. The rectangular plate contained half-strength MS agar medium; the petri dish contained TS agar broth (Sigma Aldrich). The rectangular plate and 35 mm petri dish shared common air space so that the plant seedlings were exposed to the VOCs released from the 35 mm petri dish without direct contact with the bacteria. Five-day-old *Arabidopsis* seedlings were transplanted to the rectangular plate equipped with the petri dish and were further cultured for a designated period with or without the presence of a bacterial culture in the petri dish. The distance between the seedlings and the rim of the petri dish were 1–3 cm, 4–6 cm, and 7–9 cm for the ‘proximal’, ‘mid’, and ‘distal’ positions, respectively. At the start of the bioassay, TS broth was inoculated with 50 µL of liquid culture of *B. atrophaeus* ATCC9372, which was pre-cultured overnight in 3 mL of TS liquid medium and diluted 4-fold with fresh TS liquid medium prior to bioassays. For A. bacterium and E. coli cultures, LB broth was used. For bacterial extracts or standard compounds, the sample was spotted onto a round filter paper instead of TS broth in the 35 mm petri dish, and 5 seedlings of *Arabidopsis* were transplanted to the petri dish at a distance of 1–3 cm. Bioassays were performed for a designated period of time.

The bioassays with chemical standards were basically performed as follows unless otherwise noted. Each compound was diluted to 10 mM with MeOH, and 100 µL was spotted onto filter paper that was placed in 35 mm dish; three *Arabidopsis* seedlings were pre-grown under the long-day condition without chemical treatment and were transplanted to the proximal position. Relative activity (%) was calculated as the relative values of the difference in the seminal root length of three seedlings between chemically treated and no chemical treatment control compared with that of IVA-treated seedlings.

### 2.4. Purification of Bacillus Volatile Compounds Responsible for Plant Growth Inhibition

The liquid culture of *B. atrophaeus* ATCC9372 (3 mL/plate) that was cultured overnight at 27 °C was transferred to a TS agar medium in 300 petri dishes (diameter 150 mm). After 3 days of incubation at 27 °C, the *Bacillus* cells were harvested and washed twice with distilled water by successive centrifugation at 5000× *g* for 10 min at 4 °C and sonication in 1000 mL of distilled water. The extract was centrifuged at 10,000× *g* for 15 min, and the resultant supernatant was subjected to four chromatography steps as described below. Chromatography on HP-20: The crude extract of *Bacillus* was loaded onto an open column packed with 300 mL of HP-20 (Supelco) and was separated by eluting one column volume of solvent in each step. After the initial collection of the column effluent, the column was washed once with distilled water, followed by wash-1 and wash-2 with 50% methanol and 100% methanol, respectively. The fractions were acidified with 20 mM of sulfuric acid, extracted with methylene chloride, dried with anhydrous Na_2_SO_4_, concentrated by distillation in vacuo, and reconstituted in 1 mL of 100% methanol. We spotted 10 µL from each fraction onto filter paper used in the bioassay with *Arabidopsis* seedlings. Chromatography on polyamide-6: The flow-through fraction from the HP-20 column (285 mL) was fractionated on a 300-mL bed of polyamide-6 column by flush column chromatography with increasing methanol concentration. The four fractions of flow-through, wash, elution-1 (with 50% methanol), and elution-2 (with 100% methanol) were obtained as described above. The fractions were extracted, concentrated, and reconstituted in 5 mL of 100% methanol, as described in the step using HP-20 column, and 10 µL of each fraction was used for bioassay. Chromatography on ODS: The 35/65 methanol/methylene chloride fraction from the silica gel column was further concentrated to 100 µL and fractionated by HPLC using a Cosmosil AR-II C18 column (Nacalai Tesque); the gradient elution started from 15% acetonitrile with 0.1% trifluoroacetic acid (TFA) to 85% acetonitrile with 0.1% TFA in 15 min at a flow rate of 1 mL/min. The peaks were detected by a photodiode array at 205 nm. A 100-µL aliquot from each fraction was concentrated to 20 µL and used for the bioassay. The above-described procedures for fractionation were repeated twice to test the reproducibility.

### 2.5. GC–MS Analysis

A 100-µL aliquot from the HPLC fractions was spotted onto filter paper in a 35 mm petri dish in a rectangular plate containing half-strength MS medium. The volatilized compound (atmospheric pressure at 23 °C) was trapped during overnight incubation with 2 discs of monolithic MonoTrap DCC18 (GL Science, Tokyo, Japan), which were placed at a distance of 2 cm from the 35 mm petri dish. The trapped compounds were extracted with methylene chloride, dried with anhydrous Na_2_SO_4_, and concentrated to 10 µL. We used 1 µL for the GC–MS analysis via a 6890 A with 5973 network mass selective detector (Agilent Technologies) equipped with a GC column DB1701 (model 122-0732) (Agilent Technologies, Santa Clara, CA, USA) under split mode (split ratio 1:10), with the oven temperature program starting from 10 min holding at 40 °C, followed by a gradient temperature increase to 220 °C in 30 min at a rate of 5 °C/min. The identity of the compounds was evaluated from retention time and fragmentation patterns using the Wiley 275 library. A GC column Cyclodex-B (model 112-2532) (Agilent Technologies) was used for the chiral GC–MS analysis.

### 2.6. Quantification of Short-Chain Fatty Acids Released from Bacillus Culture

For quantifying short-chain fatty acids from *Bacillus* cultures on TS medium, a 35 mm petri dish containing TS medium was placed on a rectangular plate filled with 40 mL of 1 M sodium bicarbonate. *Bacillus* was cultured for 4 days in the 35 mm petri dish, and the VOCs released from the *Bacillus* culture was extracted from the sodium bicarbonate solution as follows. The sodium bicarbonate solution was supplemented with 50 μL of 100 μM pivalic acid as an internal standard and was washed three times with 40 mL of methylene chloride. After acidification with 40 mL of 1 M sulfuric acid, the aqueous solution was extracted three times with 40 mL of methylene chloride, dried with anhydrous Na_2_SO_4_, and concentrated to 50 µL; we used 1 µL for GC–MS analysis via a 6890 A with 5973 network mass selective detector (Agilent Technologies) equipped with a GC column HP5-MS (Agilent Technologies,) under split mode (split ratio 1:10), with the oven temperature program of 10 min holding at 40 °C, followed by a gradient temperature increase to 220 °C in 45 min at a rate of 4 °C/min, and 30 min holding at 220 °C. For quantitating short-chain fatty acids from *Bacillus* in soil, a 150 g portion of experimental soil medium Metromix 350 (Sun Gro Horticulture, Agawam, MA, USA) was mixed with 0.7 g of TS broth and 120 mL of distilled water, and the mixture was autoclaved and irradiated under ultraviolet light for 30 min prior to the inoculation. The autoclaved soil inoculated with *Bacillus* inoculation was transferred to autoclaved glass petri dishes (diameter 15 cm). A glass petri dish (diameter 9 cm) filled with 80 mL of 1 M of sodium bicarbonate solution was placed on top of the soil to absorb volatile compounds with any acidity. The petri dish was covered with a lid and incubated for up to 48 h. The sodium bicarbonate solution was supplemented with 20 mM of sulfuric acid and extracted twice with 80 mL of methylene chloride. The extracts were dried with anhydrous Na_2_SO_4_, concentrated under atmospheric pressure, and reconstituted with 20 µL of methanol. A 1-µL aliquot of the concentrate was used for the GC–MS analysis on an HP5-MS column with the oven temperature program used for the analysis for quantitating the short-chain fatty acids from *Bacillus* cultures in the TS medium.

### 2.7. Bioassay Using ^14^C-Labeled IVA

In order to quantitate the amount of IVA absorbed in the tissues of *Arabidopsis* seedlings upon bioassays, ^14^C-labeled IVA was used for the bioassays, and the radioactivity from *Arabidopsis* tissue was quantified. Briefly, 10.1 µL of ^14^C-labeled IVA corresponding to a 2 mM concentration (American Radiolabeled Chemicals) was mixed with 9.9 µL of 200 mM non-radiolabeled IVA to make 100 mM of ^14^C-labeled IVA (corresponding to 50 µCi/mL) and was serially diluted with MeOH to prepare 10-mM and 1-mM solutions. Fifteen *Arabidopsis* (Col-0) seedlings (5-day-old) were transferred to test plates, and ^14^C-labeled IVA mixed with non-labeled IVA was spotted onto the filter paper in a 35 mm dish and incubated for 3 days under a long-day photoperiod at 23 °C. The seedlings were transferred to filter paper and wrapped with plastic film. Imaging plates (Fujifilm, Tokyo, Japan) were exposed for 24 h and scanned with Typhoon FLA-9500 (GE Healthcare, Chicago, IL, USA) and the radioactivity was analyzed and quantitated using Image Quant (GE Healthcare) software. Radioactivity from five seedlings either at the proximal, mid, or distal position were pooled and quantitated. Arbitral units of the radioactivity were converted to DPM by a standard curve. The experiments were repeated at least three times, independently.

### 2.8. Statistical Analysis

Statistical analysis was performed with KyPlot 6.0 (Kyenslab, Tokyo, Japan).

## 3. Results

### 3.1. The Growth of Arabidopsis Seedlings Was Inhibited by Bacillus-Derived BVOCs

**Figure 1:**
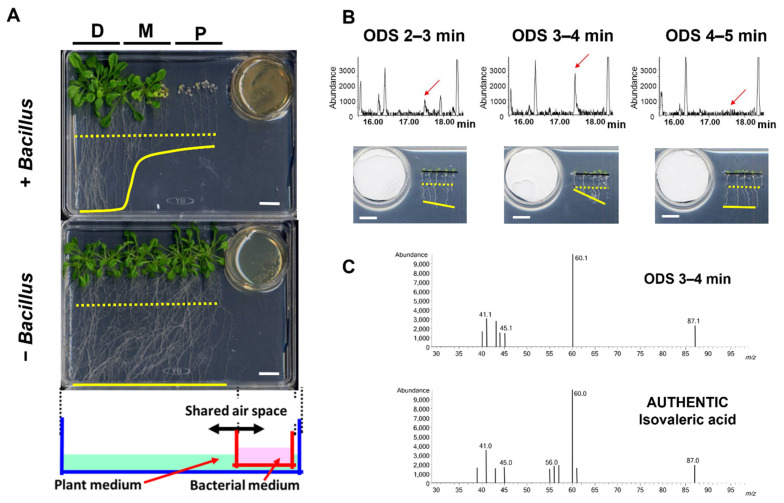
Identification of isovaleric acid as the VOC that inhibits *Arabidopsis* seedling growth. (**A**) The growth of *Arabidopsis* (Col-0) seedlings was substantially affected by volatile compounds released from the *Bacillus* culture. P, M, and D represent the relative distance between the *Arabidopsis* seedlings and the *Bacillus* culture: P; proximal, M; mid, D; distal. Shown at the bottom is a schematic of the bioassay setup. The dotted and solid yellow lines represent the positions of root tips at Day 0 and 14 of the bioassays, respectively. White bars, 1 cm. (**B**) The GC analysis and bioassay results of the representative fractions of *Bacillus* culture containing growth-inhibiting activity towards *Arabidopsis* seedlings as obtained from an ODS column chromatography. Red arrows: peaks corresponding to plant growth inhibiting activity. The dotted and solid yellow lines in the photographs represent the positions of root tips at Day 0 and 3 of the bioassays, respectively. White bars, 1 cm. (**C**) The GC–MS analysis of the peak corresponding to the plant growth inhibition activity from the fraction ODS 3–4 min as shown in (**B**) and the authentic standard of IVA.

**Figure A1:**
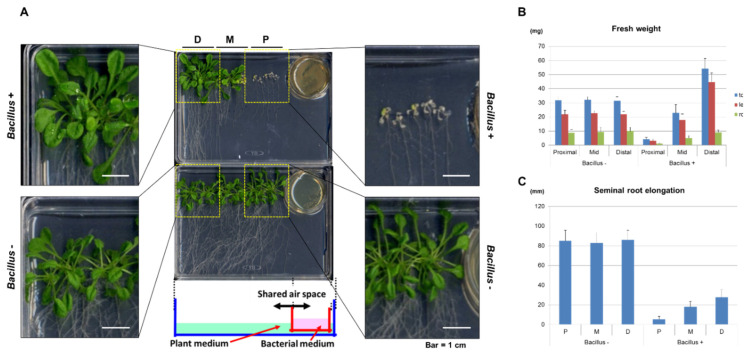
*Bacillus* VOCs affect the growth of *Arabidopsis* seedlings in a distant-dependent manner. (**A**) Magnified view of *Arabidopsis* seedlings that were subjected to the co-culture with *Bacillus* culture as shown in Figure 1. (**B**) Mean fresh weight (±SD) of the *Arabidopsis* seedlings with or without exposure to *Bacillus* VOCs for 5 days in the bioassay as shown in Figure 1A. Five seedlings each (fifteen seedlings in total) were transplanted to the proximal (P), mid (M) and distal (D) positions. Note that the fresh weight of the *Arabidopsis* seedlings was severely decreased in the P position, whereas seedlings in the D position exhibited increased biomass. (n = 5) (**C**). The mean length of seminal root elongation of *Arabidopsis* seedlings upon exposure to *Bacillus* VOCs. (n = 5).

We established a model bioassay system for co-culturing *Arabidopsis* seedlings with *B. atrophaeus* Nakamura (ATCC9372) to identify BVOCs that affect plant growth; we used a half-strength Murashige–Skoog (MS) agar medium and tryptic soy agar medium for *Arabidopsis* and *B. atrophaeus,* respectively (Figure 1A). The result showed that the seedling growth was substantially inhibited when co-cultured with a *Bacillus* culture (Figure 1A and Figure A1). The average fresh weight of total seedlings grown proximal to the *Bacillus* culture was less than 14% compared with *Arabidopsis* seedlings without a *Bacillus* co-culture (Figure A1B). The length of the seminal root was also severely inhibited (Figure A1C). These results demonstrated that the *Bacillus* culture emitted BVOCs that inhibited the growth of *Arabidopsis* seedlings. In clear contrast, the fresh weight of the seedlings showed no apparent growth inhibition but rather a slight increase in the biomass when grown distal to the *Bacillus* culture (Figure A1B). As the growth inhibition of the *Arabidopsis* seedlings was likely the direct consequence of their exposure to *Bacillus* BVOCs, we focused on the growth-inhibitory effects of *Bacillus* towards *Arabidopsis* seedlings and have tried to identify the volatile compounds responsible for this activity.

### 3.2. Bioassay-Guided Fractionation and Identification of IVA as a BVOC That Is Responsible for the Plant Growth Inhibition

**Figure A2:**
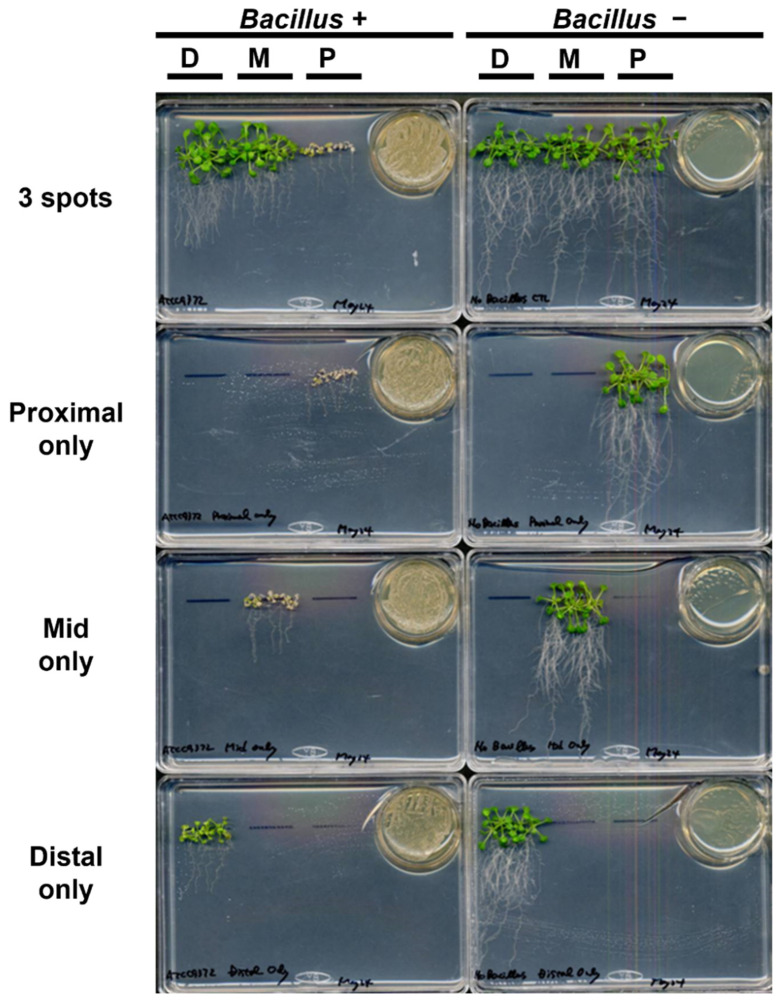
Growth alteration of the *Arabidopsis* seedlings upon exposure to VOCs of *Bacillus* culture was affected by the seedlings being relatively closer to the source of the VOCs. Either fifteen (3 spots) or five (either proximal, mid, or distal only) *Arabidopsis* seedlings were exposed to *Bacillus* VOCs for 14 days. When five *Arabidopsis* seedlings were grown in the mid position, the growth of the seedlings were severely inhibited compared with the case when the *Bacillus* was not co-cultured. Likewise, when grown only in the distal position, the inhibition of the *Arabidopsis* seedlings growth was evident compared with the no *Bacillus* control. Moreover, the growth of the seedlings grown either in the proximal, mid or distal only positions was severely inhibited by the *Bacillus* VOCs compared with the case when fifteen *Arabidopsis* seedlings were grown (3 spots). D: distal; M: mid; P: proximal.

**Figure A3:**
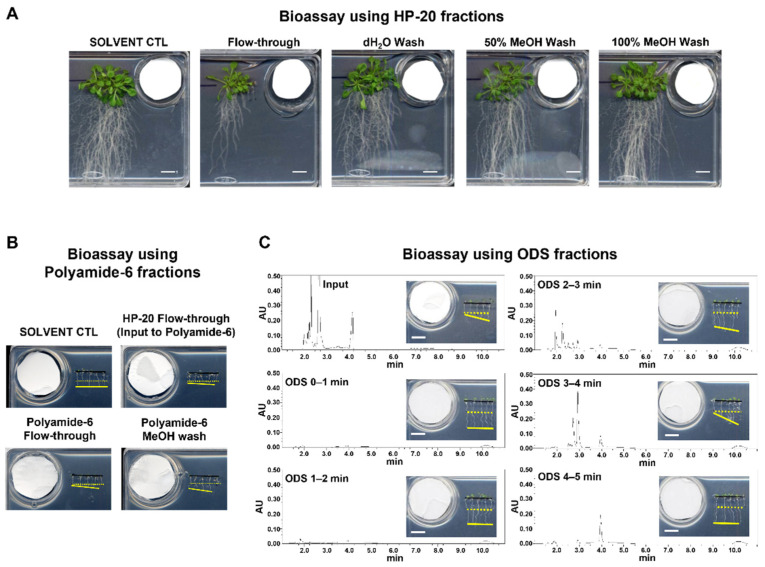
Bioassay-based purification of the compounds responsible for plant growth-inhibitory activity. All of the bioassays were conducted at 23 °C under long-day photoperiods. Solid and dashed yellow lines within inlets indicate the position of the root tips upon transplantation and after 2 days of incubation, respectively. White bars, 1 cm. (**A**) Bioassays using HP-20 fractions. Each fraction was spotted onto filter paper used in the bioassay with *Arabidopsis* seedlings. Photographs were taken after 3 weeks of incubation. White bars; 2 cm. (**B**) Bioassays using polyamide-6 fractions. The flow-through fraction from the HP-20 column was fractionated on a polyamide-6 column with increasing methanol concentration. For the bioassays, 10 µl out of 5 ml of each fraction was used. Photographs were taken after 2 days of incubation. (**C**) Bioassay testing fractions from ODS chromatography. Ultraviolet absorbance at 205 nm was monitored by a photodiode array detector.

To identify the volatile compounds with growth-inhibiting activity towards *Arabidopsis* seedlings, we performed a bioassay-based 3-step purification (HP-20, Polyamide-6, and ODS column chromatography), using an aqueous crude extract from *B. atrophaeus* cells as the starting material. An inhibition of the seminal root length of *Arabidopsis* seedlings was monitored as the prime index of growth inhibition during the bioassay-guided purification of BVOCs. Fractionation of the crude extract with HP-20 and the following bioassay experiments indicated that the elongation of seminal roots of *Arabidopsis* seedlings was inhibited when exposed to the flow-through fraction (Figure A2A). The flow-through fraction of the HP-20 column chromatography was then subjected to fractionation with polyamide-6 resin, and the flow-through and MeOH as fractions were obtained. The bioassay experiments using the polyamide-6 fractions showed that the flow-through fraction contained the plant growth-inhibiting activity (Figure A2B). The flow-through fraction from polyamide-6 fractionation was further subjected to ODS fractionation, followed by bioassay experiments. The results indicated that the seminal root elongation of *Arabidopsis* seedlings was inhibited when exposed to the fraction ‘ODS 3–4 min’ (Figure A2C). Finally, each ODS fraction including ‘ODS 3–4 min’ was analyzed by a gas chromatography–mass spectrometry (GC–MS) analysis. The GC–MS analysis of the extract active fraction (ODS 3–4 min) identified that a fragmentation pattern of the peak correlating well with the activity matched with the isovaleric acid (IVA) standard (Figure 1B,C). Co-chromatography analysis with the authentic IVA standard further supported the notion that the volatile compound responsible for the growth-inhibiting activity of the *Arabidopsis* seedlings was IVA (Figure A3).

### 3.3. The Amount and Tissue Distribution of IVA Incorporated in Arabidopsis Seedlings

**Figure 2:**
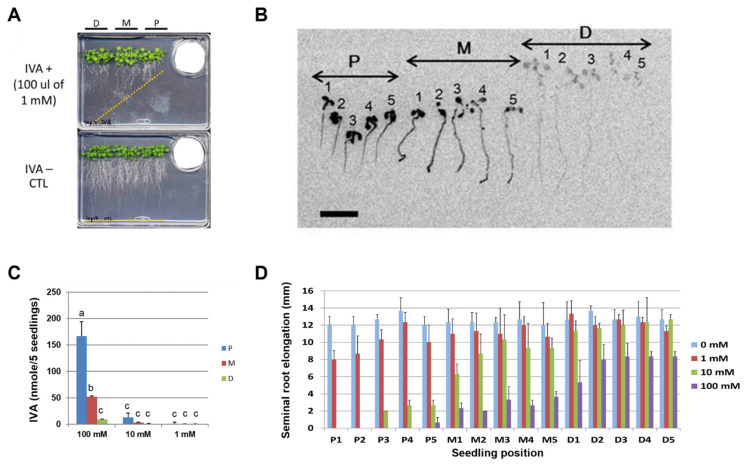
IVA exhibits growth-inhibitory activity towards *Arabidopsis* seedlings. P: proximal; M: mid; D: distal. (**A**) The elongation of the *Arabidopsis* seminal root was inhibited by the vapor of the IVA standard. The standard solution of IVA was spotted onto filter paper placed in a 35 mm dish, and the *Arabidopsis* seedlings were subjected to the bioassay. Orange dotted line: positions of root tips on Day 14 of the bioassay. (**B**) Autoradiogram of the *Arabidopsis* seedlings pre-cultured without IVA for 5 days and exposed to the vapor of 100 μL of 10 mM IVA (a mixture of non-radiolabeled and ^14^C-labeled IVA) that was spotted onto filter paper in a 35 mm dish. The *Arabidopsis* seedlings were imaged after 3 days of co-culturing. The numbers represent the position of each seedling relative to 35 mm dish. Bar = 1 cm. (**C**) The amount of ^14^C-labeled IVA absorbed per five seedlings was quantified by a phosphor imager, 1–100 mM; The concentrations of ^14^C-labeled IVA solutions used in the assay. Different letters indicate significant differences according to Tukey’s means comparison test (*p* ≤ 0.05). Error bars represent the SD. (n = 3) (**D**) Seminal root elongation of the *Arabidopsis* seedlings after exposure to ^14^C-labeled IVA. P1–P5, M1–M5, and D1–D5 correspond to the position of each seedling relative to the source of ^14^C-labeled IVA. Different concentrations of IVA (0, 1, 10, and 100 mM) were used in the experiment. The error bars represent the SD. (n = 3). P: proximal position; M: mid position; D: distal position.

In order to quantify the amount and distribution of IVA in the *Arabidopsis* seedlings, *Arabidopsis* seedlings were exposed to the vapor of ^14^C-labeled IVA, and the radioactivity absorbed in *Arabidopsis* tissue was quantified with a phosphor imager. The data indicate that the elongation of the seminal root was inhibited upon exposure to IVA (Figure 2A). The result phenocopied the apparent inhibition of seminal root elongation by the BVOC that was released from *Bacillus* spp. (Figure 1A). The result further showed that the radioactivity was detected from the whole part of the seedlings, showing a preference for cotyledons in seedlings in the proximal position and for root tips in seedlings in the mid position (Figure 2B). Moreover, a higher level of radioactivity was detected from seedlings at the proximal compared to the distal position. Specifically, 10- and 3-times higher levels of radioactivity from ^14^C-labeled IVA were detected in the seedlings in the proximal and mid position, respectively, compared with the distal position when exposed to 100 mM of IVA containing ^14^C-labeled IVA (Figure 2B). Seminal root elongation of *Arabidopsis* seedlings was inhibited in a manner dependent on the distance from the source of the IVA vapor as well as the concentration of IVA solution. Elongation of seminal roots of the seedlings at the Distal position was less inhibited compared to that at the Mid position, and the seedlings at the Proximal position were even more severely inhibited. In addition, seminal root elongation was more inhibited when a higher concentration of IVA solution was used (Figure 2C). Furthermore, we conducted bioassays using ^14^C-labeled IVA under different light conditions and tested whether the light condition affects the growth inhibition of *Arabidopsis* seedlings by the vapor of IVA (Figure A5). Notably, the radioactivity in *Arabidopsis* seedlings was higher when grown under long-day photoperiod as compared with continuous dark during the bioassay; the amount of ^14^C-labeled IVA levels that were detected in *Arabidopsis* seedlings were 5- to 8-times higher in light-grown seedlings compared with dark-grown seedlings. The data indicate that the incorporation of ^14^C-labeled IVA primarily occurred under photosynthetic conditions. Altogether, the results revealed that exogenously applied IVA is incorporated into *Arabidopsis* seedlings by the cotyledons and at least in part by the roots rather than being submissively attached to the surface of the seedling tissues, which results in a growth inhibition of *Arabidopsis* seedlings in planta.

### 3.4. Structure–Activity Relationship of IVA and Its Related Compounds

**Figure A4:**
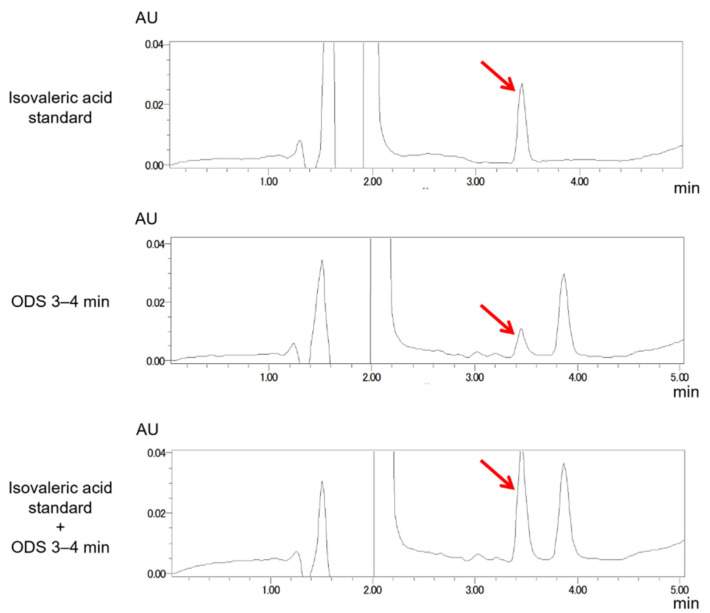
A peak in the fraction ODS (3–4 min) exhibiting plant growth inhibiting activity co-chromatographed with the IVA standard. A peak in the HPLC chromatogram of the fraction ODS (3–4 min) with plant growth inhibition activity (red arrows) was co-chromatographed with that of the authentic standard of isovaleric acid.

Further chiral GC–MS analysis of the active fraction from the ODS column chromatography (ODS 3–4 min) revealed that (*S*)-2-methylbutyric acid, a structural isomer of IVA, was also found in *Bacillus* crude extracts, although to a much lesser extent compared with IVA (Figure A4). We therefore examined the plant growth-inhibiting activity of (*S*)-2-methylbutyric acid using the authentic standard and found that (*S*)-2-methylbutyric acid exhibited a lower but substantial level of plant growth-inhibition activity relative to IVA. However, together with the fact that the amount of (*S*)-2-methylbutyric acid detected in the active fraction was negligible compared with that of IVA, we concluded that IVA was primarily responsible for the plant growth inhibiting activity of *Bacillus* BVOCs.

**Figure 3:**
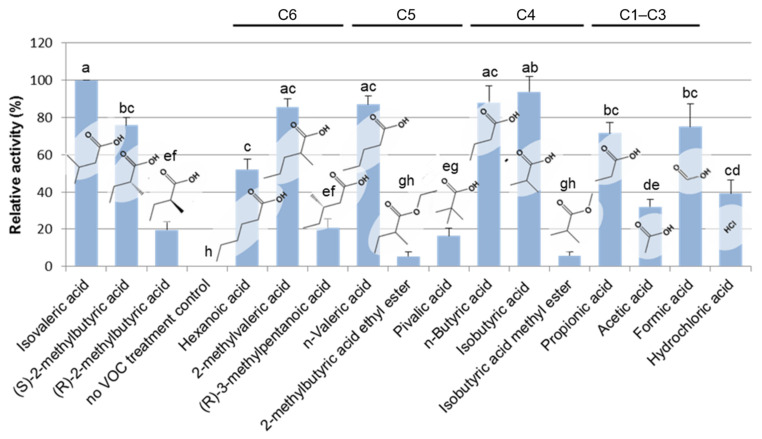
Structure–activity relationship of IVA and its analogs. Various structural analogs of IVA were subjected to the bioassay for detecting plant growth-inhibiting activity by monitoring the inhibition of seminal root elongation. The inhibitory activity of seminal root elongation exhibited by each chemical was evaluated after 5 days of incubation and presented as values relative to that of isovaleric acid. It is notable that (*R*)-2-methylbutyric acid showed significantly lower activity of plant growth inhibition compared with (*S*)-(+)-2-methylbutyric acid, which is one of the two major BVOCs among *Bacillus* BVOCs. C1–C6 represents the number of carbon atoms in the main chain length of each compound. Mean values (± SD) of the plant growth inhibition activity of each standard chemical that are represented as the relative activity to that of IVA. Different letters indicate significant differences according to Tukey’s means comparison test (*p* ≤ 0.01). The error bars represent the SD. (n = 3).

To analyze the structure–activity relationship of short-chain fatty acids towards *Arabidopsis* seedling growth, various structural analogs of IVA were exogenously applied to *Arabidopsis* seedlings, and their activities were tested by monitoring the inhibition of the seminal root elongation (Figure 3). Most monocarboxylic acids with the main chain length being shorter than seven carbons, namely hexanoic, pentanoic, butyric, propionic, and formic acids, as well as the selected isomers of those acids clearly showed plant growth-inhibitory activity at the level comparable to that of IVA. In contrast, hydrochloric, acetic acid, 3-methylpentanoic acid, and non-natural pivalic acid showed a moderate activity level of plant growth-inhibition compared with IVA. Moreover, 2-methylbutyric acid ethyl ester and isobutyric acid methyl ester did not show significant plant growth inhibitory activity. These data clearly indicate the requirement of the free carboxyl group and the main chain length comprise four to five carbons for the analogs to exhibit plant growth-inhibiting activity at a level equivalent to IVA. Notably, (*R*)-2-methylbutyric acid, an enantiomeric isomer of (*S*)-2-methylbutyric acid, showed significantly lower activity of plant growth-inhibition compared with (*S*)-2-methylbutyric acid, which is one of the two major BVOCs among *Bacillus* BVOCs.

## 4. Discussion

Numerous reports suggest that BVOCs might play significantly diverse roles in the interspecific as well as interkingdom interaction between various organisms, including but not restricted to, microbe–microbe and microbe–mammal interactions. For example, gut microbes produce butyrate that triggers macrophage-mediated immune responses under anaerobic conditions [16]. Moreover, 2-aminoacetophenone produced by *Pseudomonas aeruginosa* induces insulin resistance, thus modulating the metabolism of host cell [17]. For microbe–microbe interaction, benzaldehyde, 1,2-benzisothiazol-3(2*H*)-one, and 1,3-butadiene released from *Bacillus* spp. have been shown to inhibit the growth of *Ralstonia solanacearum*, which causes bacterial wilt disease in tobacco plants [18]. These reports collectively propose that BVOCs exhibit various biological activities against a wide range of prokaryotic and eukaryotic species. Therefore, from a biotechnological point of view, the identification of an array of BVOC-releasing microorganisms provides new tools that allow for the manipulation of plant growth and development. Previous studies on the physiological effects of BVOC on plants often focused on microbial strains that confer positive rather than negative impacts upon plant growth and development. Accordingly, whether and how BVOCs regulate the development, immunity, and resilience to stress environments have been recently studied [19]. The report jointly shows that the changes in the level of various phytohormones and the expression of their biosynthetic enzyme genes partly explain the physiological responses of plants upon exposure to BVOCs. In contrast, less is known about the molecular mechanism of how BVOCs inhibit normal plant growth.

**Figure A5:**
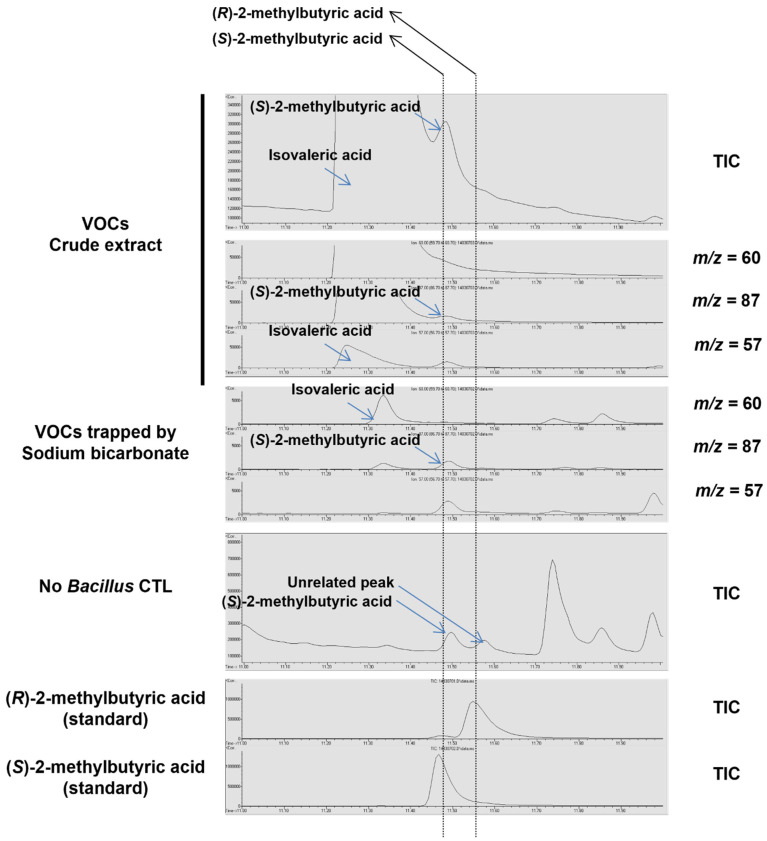
Chiral GC–MS analysis on VOCs from the fraction exhibiting plant growth-inhibiting activity of the ODS column chromatography. The crude extract of *Bacillus*-derived VOCs and *Bacillus* VOCs trapped by sodium bicarbonate was subjected to the chiral GC–MS analysis. Note that (*S*)-2-methylbutyric acid was detected along with IVA from the two samples, but not (*R*)-2-methylbutyric acid. TIC: total ion content.

**Figure A6:**
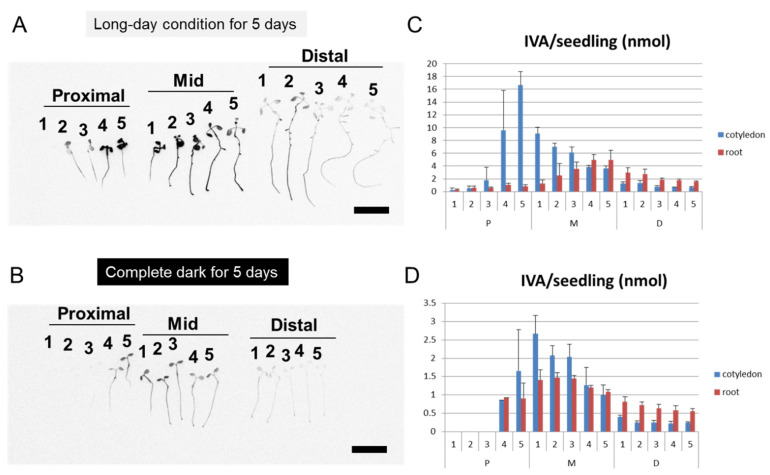
Light condition affects the incorporation rates of ^14^C-labeled IVA into the *Arabidopsis* seedlings. Bars: 1 cm. Numbers represent individual seedlings. (**A**) Autoradiogram of the *Arabidopsis* seedlings that were subjected to the bioassay ^14^C-labeled IVA under the long-day photoperiod for 5 days. (**B**) Autoradiogram of the *Arabidopsis* seedlings that were subjected to the bioassay ^14^C-labeled IVA under the complete dark condition for 5 days. (**C**) Radioactivity detected either from the cotyledons or the roots of the *Arabidopsis* seedlings from the bioassay in (**A**). (**D**) Radioactivity detected either from the cotyledons or the roots of the *Arabidopsis* seedlings from the bioassay in (**B**).

Through a bioassay-guided purification, we identified that IVA contained in the BVOCs of *Bacillus* spp. is responsible for the inhibition of seminal root elongation of the *Arabidopsis* seedlings in our co-culturing system (Figure 1, Figure A2 and Figure A3). IVA is known to be readily soluble to water (http://www.chemindex.com/ accessed on 11 July 2022.) yet volatile as shown by the standard solution in Figure 2 and Figure A5 and by *Bacillus* spp. in Figure A6. These results collectively show that IVA was released from *Bacillus* spp. as a volatile compound and was absorbed by the *Arabidopsis* seedlings. Furthermore, we identified that (*S*)-2-methylbutyric acid, a structural isomer of IVA, exists as a minor component in a crude extract of *Bacillus* cells (Figure A4) and also exhibits plant growth inhibitory activity comparable to IVA (Figure 3). IVA and (*S*)-2-methylbutyric acid are short-chain fatty acids that are likely derived from their CoA esters either through hydrolysis or the activities of phosphotransacylase and acyl kinase enzymes [20,21,22]. Isovaleryl-CoA and 2-methylbutyryl-CoA are intermediates in the degradation pathway of the branched-chain amino acids (BCAAs) leucine and isoleucine, respectively, and are known to ubiquitously occur in an array of microbial species. The result therefore suggests that various bacterial species other than *Bacillus* spp. might also be capable of producing IVA or its structural analogs through the BCAA degradation pathway and thus inhibit plant growth under appropriate conditions.

Because both IVA and (*S*)-2-methylbutyric acid are derived from their respective CoA esters, the level of BCAA degradation pathway intermediates released from soil microbes should correlate with the plant growth-inhibiting activity. Interestingly, the *Arabidopsis* mutant that lacks functional isovaleryl-CoA dehydrogenase (IVD) that is involved in leucine degradation pathway was more sensitive to prolonged dark treatment, where sucrose and other sugars rapidly decline [23]. Moreover, *ivd* mutant plants accumulated a higher amount of isovaleryl-CoA compared with wild-type plants under extended dark conditions. Therefore, the malfunction of the BCAA degradation pathway can lead to severe growth retardation under stress conditions with a limited level of sugars. Further studies will uncover whether an exogenous application of IVA affects the metabolic pathway of BCAAs in plants.

In the co-culture experiments of the *Arabidopsis* seedlings and *Bacillus* spp., we observed that the *Arabidopsis* seedlings grown in the distal position exhibited a slight growth promotion rather than inhibition (Figure A1). On the other hand, the growth promotion of plants in the distal position by IVA treatment (Figure 2A) was not evident as in the case with *Bacillus* BVOCs. Likewise, the growth inhibition in cotyledons by IVA was not as evident as in the case of BVOCs (Figure 1A). These observations collectively suggest the involvement of unknown chemical components other than IVA for inducing the full spectrum of growth alteration that was observed in the *Arabidopsis* seedlings when co-cultured with *Bacillus* spp. (Figure 2). Our bioassay experiments using ^14^C-labeled IVA showed that the radioactivity was detected from the whole tissue of the *Arabidopsis* seedlings (Figure 2). Because the level of radioactivity was mostly higher in cotyledons than in seminal roots, cotyledons are the major tissue of IVA accumulation in our experimental setup. Interestingly, the cotyledons of the *Arabidopsis* seedlings grown under the long-day photoperiod accumulated higher levels of exogenously applied ^14^C-labeled IVA compared with those under the complete dark condition (Figure A5). Within the root tissue, on the other hand, root tips are the primal sites of the radioactivity. In addition, as in the case of cotyledon, the radioactivity detected by the root is higher in the *Arabidopsis* seedlings grown under the long-day photoperiod than in those under the complete dark condition (Figure A5). These data indicate that the incorporation of ^14^C-labeled IVA primarily occurred under photosynthetic conditions, which correlates with the degree of stomata closure and water uptake [24]. It is noteworthy that, under long-day photoperiod, the level of radioactivity in cotyledons was highest at the proximal position, whereas that in the roots was at the mid position (Figure A5). Although to a lesser extent, a similar trend in the radioactivity was observed from the *Arabidopsis* seedlings under the complete dark condition. The position of the *Arabidopsis* seedlings with the highest level of radioactivity in the cotyledons was relatively closer to the source of the exogenously supplied ^14^C-labeled IVA than the position where the level of radioactivity in the roots was the highest. These results predict either the existence of a molecular mechanism that allocates exogenously supplied IVA and/or its metabolites across the cotyledon and root.

Among other plant tissues, the root is likely the prime site of contact with BVOCs that were released from soil microbes. Therefore, it is plausible that the inhibition of seminal root elongation by IVA is triggered by the uptake of IVA in root rather than by the allocation of IVA that was originally incorporated in arial parts of the plant. On the other hand, because BVOCs have been known to be released into the atmosphere [25,26], it is also possible that cotyledons are exposed to IVA and other BVOCs derived from soil microbes. A future development of the experimental conditions that allows for the discrimination of the effects of IVA on the above-ground and below-ground parts of the plant will facilitate a better understanding on the biological activity of BVOCs.

Plants release a plethora of volatile compounds in their above-ground part and not only use them to interact with pollinators, herbivores, microorganisms, and various other organisms, but also between plants themselves [27,28]. Recent studies have shown that volatile alcohols are stored in plant tissues as non-volatile glycosides, often at higher levels than free alcohols, which are produced by UDP-glycosyltransferases (GTs). For example, *Vitis vinifera* (grape) accumulates various terpenoids in its berry as their respective glycosides [29]. Notably, VvGT7 alleles obtained from various grape cultivars with characteristic flavor profiles show different substrate preferences towards terpene alcohol, phenols, and short-chain alcohols. Moreover, VvGT7 preferentially glucosylated (*R*)-citronellol was comparable to (*S*)-citronellol in an enantioselective manner. In addition, grape leaves and berries have been shown to accumulate exogenously applied phenols and other volatile compounds as glycosides [30]. Moreover, *Camellia sinensis* (tea) is capable of accumulating glycosidic forms of *cis-*3-hexenol derived from mechanically-damaged neighboring tea plants [31]. These reports reveal that GTs play crucial roles in selectively accumulating volatile alcohols in the above-ground parts of plants.

On the other hand, not much has been elucidated as to whether and how plants respond to BVOCs, especially with respect to short-chain fatty acids emitted by soil microbes in a specific manner. In our study, we observed that *Bacillus* crude extract that exhibited growth-inhibiting activity towards *Arabidopsis* seedlings contained IVA as a primal component and its structural isomer (*S*)-2-methylbutyric acid as a minor component (Figure A5). Although the amount detected in the extract was low, (*S*)-2-methylbutyric acid inhibited seminal root elongation at a level comparable to that of IVA when its authentic standard was exogenously supplied (Figure 3). In contrast, non-natural (*R*)-2-methylbutyric acid, which was not detected in the *Bacillus* crude extract, did not clearly inhibit seminal root elongation when exogenously supplied (Figure 3). Moreover, pivalic acid, another non-natural compound with five-carbon atoms, showed a considerably low level of inhibitory activity towards seminal root elongation. The apparent low inhibitory activity of seminal root elongation by non-natural isomers of IVA might reflect the structural requirement by the molecular mechanism of how *Arabidopsis* perceives IVA and its related BVOCs. It is notable that 2-methylbutyric acid ethyl ester did not exhibit a substantial level of inhibitory activity towards seminal root elongation (Figure 3). The result suggests that a free carboxyl group is required for 2-methylbutyric acid to inhibit the growth of *Arabidopsis* seedlings at a level comparable to IVA. Because various plant hormones with a carboxylic acid moiety and short-chain fatty acids are stored as glucose esters [32,33,34], it is plausible that IVA and (*R*)-2-methylbutyric acid are also conjugated to glucose. Alternatively, IVA and (*R*)-2-methylbutyric acid might be conjugated back with coenzyme A through the action of CoA ligases.

The discovery of a novel biological function of IVA as a bacterial volatile regulator of plant growth identifies a new mode of plant–microbe interaction through short-chain fatty acids within BVOCs that are universally expressed across the bacterial and plant kingdoms. This suggests that a further characterization of the molecular mechanism of plant–microbe interactions through IVA will shed new light on the development of screening strategy for isolating soil microbes as possible bioherbicides [35] by monitoring the emission of IVA. Furthermore, this will also highlight the versatility and commonalities of the biological significance of various other small molecules that universally exist in plants, soil microbes, and the rhizosphere.

## Figures and Tables

**Figure A7:**
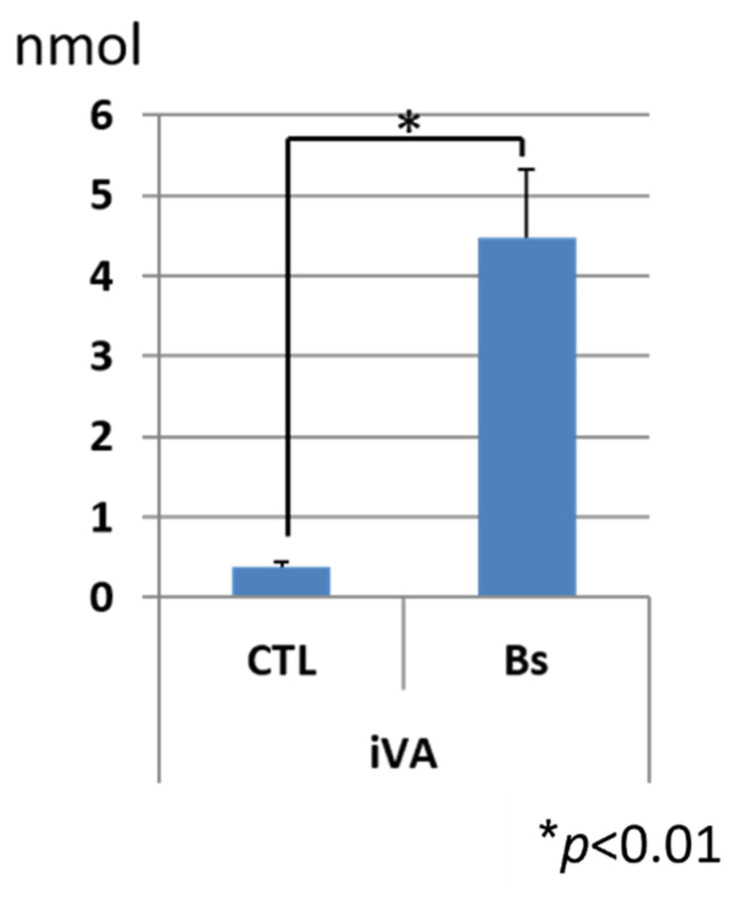
Quantification of IVA released from the *Bacillus* spp. culture. *Bacillus* spp. was cultured in a 35 mm dish with agar medium of tryptic soy broth. A 35 mm dish was covered with a rectangular petri dish containing 40 ml of NaHCO_3_. After 2 days of culture, the IVA that was released from *Bacillus* and absorbed in NaHCO_3_ was extracted twice with equal volume of methylene chloride, concentrated in a hot water bath (40 degrees), and was subjected to GC–MS analysis. The calculation was based on the corresponding peak area of a fragmentation ion of IVA in a mass chromatogram (*m/z* 87). CTL: control experiment with no *Bacillus* culture; Bs: experiment with *Bacillus* culture. (n = 3).

## Data Availability

The data presented in this study are available in the main article.
